# Case report: Middle cranial fossa arachnoid cyst in association with subdural hygroma

**DOI:** 10.4103/0971-3026.41831

**Published:** 2008-08

**Authors:** Pranjal Goswami, Nirod Medhi, Pratul K Sarma, Bhaskar J Sarmah

**Affiliations:** Department of Radiology, Primus, GS Road, Bhangagarh, Guwahati-781005, Assam, India

**Keywords:** Arachnoid cyst, head injury, subdural hygroma

Arachnoid cysts are congenital, cerebrospinal fluid (CSF)-filled, intra-arachnoidal lesions that are a common incidental finding on routine brain imaging. While this lesion can present as a mass lesion, the vast majority is generally asymptomatic. Rarely, posttraumatic or spontaneous rupture of arachnoid cysts can result in intracystic hemorrhage, subdural hematoma, or subdural hygroma. We describe a rare presentation of an arachnoid cyst, which ruptured after a minor head injury and resulted in a subdural hygroma.

Arachnoid cysts are intra-arachnoidal space-occupying lesions containing fluid similar to CSF. They are benign in nature and congenital in origin. Arachnoid cysts represent 1% of all nontraumatic intracranial masses.[1] Most arachnoid cysts are supratentorial in location and 50-65% occur in the middle cranial fossa. Arachnoid cysts can also occur less frequently in the suprasellar and quadrigeminal cisterns, cerebral convexities, cerebellopontine angle, and cisterna magna. Occasionally, arachnoid cysts are complicated by intracystic or subdural hemorrhage, with or without history of preceding trauma.[1–3] A very rare complication of arachnoid cysts is the spontaneous or traumatic development of subdural hygromas.[2,4–7] We report a patient with an intracranial arachnoid cyst that was complicated by a subdural hygroma after a minor head injury.

## Case History

**Figure 1 (A-C):**
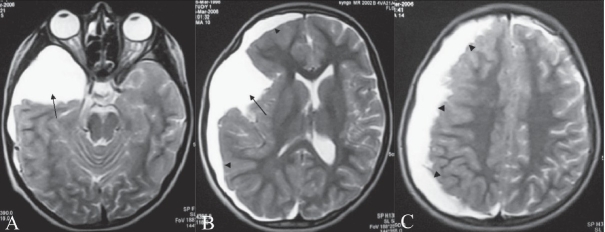
T2W axial images of the brain at different levels show a right middle cranial fossa arachnoid cyst (arrow) with an associated subdural hygroma (arrowheads)

**Figure 2 (A, B):**
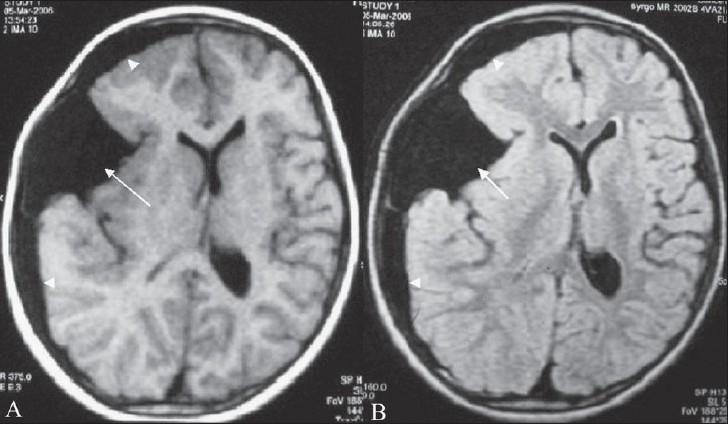
Axial T1W (A) and axial FLAIR (B) images show a hypointense arachnoid cyst (arrow) and an associated subdural hygroma (arrowhead)

An 8-year-old boy presented with a history of intermittent headache and nausea. One week prior to his presentation he had slipped while walking and suffered a mild head injury. There was no history of loss of consciousness at the time of the injury. His neurological examination was unremarkable and there was no focal neurological abnormality, cranial nerve deficit, or papilledema. He had no significant past medical history. MRI of the brain showed a right middle cranial fossa arachnoid cyst with an associated right-sided subdural hygroma, causing mass effect with ipsilateral ventricular compression and effacement of the convexity sulcal spaces [Figures 1 and 2]. The patient underwent surgery, and cyst fenestration and evacuation of the right-sided subdural hygroma were performed. His symptoms resolved immediately and he made an uneventful recovery.

## Discussion

Most arachnoid cysts are asymptomatic and may not produce any symptoms throughout life. They are generally diagnosed incidentally on CT scan and MRI. Symptoms are produced when arachnoid cysts are large or are complicated by subdural hematoma or intracystic hemorrhage. The bleeding is probably caused by the disruption of cortical veins that frequently traverse the cyst near its periphery.[2] A case of cerebral aneurysm rupturing into an arachnoid cyst, with formation of an adjacent acute subdural hematoma, has also been reported.[8]

Rarely, arachnoid cysts may become symptomatic due to the development of a subdural hygroma following traumatic or spontaneous rupture.[1–7] Appreciation of this complication has implications in the management of these patients, because there is a possibility of reaccumulation of cyst fluid in the subdural compartment despite burr hole drainage of the collection; this is due to the possibility of a communication developing between the arachnoid cyst and the subdural compartment following rupture. Because of this reason, there is a rationale for inserting a drain for immediate definitive treatment. An MRI will help to distinguish between acute or subacute hemorrhage and subdural hygroma.

Although the precise pathogenesis of a subdural hygroma in a patient with an intracranial arachnoid cyst is not fully understood, trauma appears to be the most important causative factor.[2,4–7] This trauma is often trivial.[4–7] It is suggested that acute rupture results in both rapid fluid shift and increased production of fluid from the cyst wall.[7] The majority of arachnoid cysts complicated by subdural hygromas are located in the middle cranial fossa.[4–7]

Our patient had a relatively large middle cranial fossa cyst, which was asymptomatic prior to its rupture. The patient developed symptoms of raised intracranial pressure, following a minor head injury. Though the majority of arachnoid cysts can be managed conservatively, surgical treatment is appropriate, as in our case, when there is an associated subdural hygroma.[5,6]
